# Performance of AnMBR in Treatment of Post-consumer Food Waste: Effect of Hydraulic Retention Time and Organic Loading Rate on Biogas Production and Membrane Fouling

**DOI:** 10.3389/fbioe.2020.594936

**Published:** 2021-01-18

**Authors:** Javkhlan Ariunbaatar, Robert Bair, Onur Ozcan, Harish Ravishankar, Giovanni Esposito, Piet N. L. Lens, Daniel H. Yeh

**Affiliations:** ^1^Department of Civil and Mechanical Engineering, University of Cassino and Southern Lazio, Cassino, Italy; ^2^Department of Civil and Environmental Engineering, University of South Florida, Tampa, FL, United States; ^3^Environmental Engineering Department, Istanbul Technical University, Istanbul, Turkey; ^4^Department of Microbiology, National University of Ireland Galway (NUIG), Galway, Ireland

**Keywords:** anaerobic membrane bioreactor, food waste, hydraulic retention time, organic loading rate, biogas, membrane fouling

## Abstract

Anaerobic digestion of food waste (FW) is typically limited to large reactors due to high hydraulic retention times (HRTs). Technologies such as anaerobic membrane reactors (AnMBRs) can perform anaerobic digestion at lower HRTs while maintaining high chemical oxygen demand (COD) removal efficiencies. This study evaluated the effect of HRT and organic loading rate (OLR) on the stability and performance of a side-stream AnMBR in treating diluted fresh food waste (FW). The reactor was fed with synthetic FW at an influent concentration of 8.24 (± 0.12) g COD/L. The OLR was increased by reducing the HRT from 20 to 1 d. The AnMBR obtained an overall removal efficiency of >97 and >98% of the influent COD and total suspended solids (TSS), respectively, throughout the course of operation. The biological process was able to convert 76% of the influent COD into biogas with 70% methane content, while the cake layer formed on the membrane gave an additional COD removal of 7%. Total ammoniacal nitrogen (TAN) and total nitrogen (TN) concentrations were found to be higher in the bioreactor than in the influent, and average overall removal efficiencies of 17.3 (± 5) and 61.5 (± 3)% of TAN and TN, respectively, were observed with respect to the bioreactor concentrations after 2 weeks. Total phosphorus (TP) had an average removal efficiency of 40.39 (± 5)% with respect to the influent. Membrane fouling was observed when the HRT was decreased from 7 to 5 d and was alleviated through backwashing. This study suggests that the side-stream AnMBR can be used to successfully reduce the typical HRT of wet anaerobic food waste (solids content 7%) digesters from 20 days to 1 day, while maintaining a high COD removal efficiency and biogas production.

## Introduction

Anaerobic digestion (AD) is one of the most important and sustainable processes used for the treatment of organic solid waste (OSW). It combines pollution reduction, energy production, and nutrient recovery from OSW with limited environmental impacts (Khalid et al., 2011). Among various substrates used for AD, there is a growing interest in treating food waste (FW) due to its high generation rate and easy biodegradable characteristics (Ariunbaatar et al., 2014a). There is a strong policy intend to encourage the AD of FW as the governments in Europe have set significant targets to reduce the amount of biodegradable waste to be landfilled as well as to increase the recycling rate and energy recovery (Browne and Murphy, 2013).

AD is a biological process that converts complex substrates into biogas and digestate by microbial conversions in the absence of oxygen. Anaerobic microorganisms grow slowly, and biomass retention is one of the most important aspects of AD (Fuchs et al., 2003). It is known that the AD of FW is prone to failure at high organic loading rates (OLRs), as slow growing methanogens cannot metabolize fast enough the produced volatile fatty acids or are even washed out, resulting in an acidification of the reactor which also inhibits the methanogens (Guo et al., 2014). Therefore, to prevent acidification, larger reactor volumes and/or long HRTs are often necessary. Considering the large volumes of waste requiring treatment as well the costs of larger reactors, a more efficient reactor design is required to retain the microbial biomass in the system while maintaining a stable operation at a short HRT. This has led to the growing popularity of the anaerobic membrane bioreactor (AnMBR) configuration, which decouples the HRT from the solids retention time (SRT) (Smith et al., 2012; Stuckey, 2012).

The AnMBR offers several advantages over conventional AD processes: (i) an ability to deal with higher organic loads even at unfavorable conditions; (ii) increased production of biogas with a higher methane content; (iii) less production of sludge; (iv) better quality effluent with no pathogens and solids; and (v) reduced footprint of the AD system (Skouteris et al., 2012; Dvorák et al., 2016). In fact, the AnMBR has been highlighted as a sustainable technology for capturing resources i.e., energy and nutrients (Stuckey, 2012; Browne and Murphy, 2013). Although the performance of an AnMBR has been studied thoroughly for the treatment of various wastewaters (Dereli et al., 2012), there has been limited research on the application of AnMBR for FW directly (Lee et al., 2014; Cheng et al., 2018), with few other studies focusing on treatment of food waste coupled with domestic wastewater (Jeong et al., 2017; Cho et al., 2018; Amha et al., 2019).

Despite AnMBR being recognized as a promising technology, membrane fouling remains the “Achilles heel” of membrane processes (Lee et al., 2014; Cho et al., 2018). Membrane fouling results in flux decline, thereby increasing the overall energy requirement and decreasing the membrane's life. Common strategies to alleviate membrane fouling include cleaning, backwashing, gas sparging, membrane relaxation and through operational configurations, i.e., submerged or side-stream (Robles et al., 2018). In the side-stream configuration, the membrane is placed externally to the reactor in a re-circulation loop as compared to being submerged in the bioreactor, thereby lowering membrane fouling along with maintaining a high permeate flux (Dvorák et al., 2016).

To overcome the challenges of treating FW through AD and operational issues with AnMBR, the present work aims to study the performance of an AnMBR system in a side-stream membrane configuration to treat diluted FW. The effect of OLR and HRT on the treatment of diluted FW and biogas production was examined using a fully automated lab-scale side-stream AnMBR system that was operated for a total of 100 days. Membrane fouling was monitored through trans-membrane pressure (TMP). The study exhibited the feasibility of treating diluted FW at low HRT with a high COD removal efficiency and biogas production yield.

## Materials and Methods

### Seed Sludge and Influent

The seed sludge (i.e., anaerobic digester sludge) was obtained from the Howard F. wastewater treatment plant (Tampa, FL, United States) and used for both batch and AnMBR experiments. The synthetic FW was prepared mimicking a typical post-consumer FW according to Ariunbaatar et al. (2014b). Ingredients included meat (chicken, beef, pork and fish), cheese, bread, rice, pasta, oranges, tomatoes, potatoes, apples, eggplant, spring mix salad, and bananas. All the ingredients were blended to a homogenous pulp and stored at 4°C not more than 2 weeks. The lab-made FW was mixed and diluted with tap water in order to maintain the solids content at around 7% TS and blended prior to use as influent. This dilution of lab-made FW to 7% is within the wet AD regime (Sarker et al., 2019) and avoided the solids from clogging the tubes and pumps. Sodium bicarbonate (NaHCO_3_) was added to the influent to provide necessary (>1,500 mg/L and pH > 7.2) alkalinity. The sludge and the influent were sieved through a no. 20 mesh (0.841 mm) to remove particles that could clog the membrane tubes.

### Biomethane Potential Test

Biomethane potential (BMP) tests were conducted to estimate the highest amount of biomethane that can be produced from the FW. BMP tests were carried out in serum bottles (total volume of 120 mL) in duplicates without mixing (Ariunbaatar et al., 2014b). The food to inoculum ratio was 0.5 gVS/gVS, and all bottles were placed in a Fisher ISOTEMP incubator 200 series model 230D. To provide sufficient total alkalinity throughout the experiment, sodium bicarbonate (4.5 g NaHCO_3_/L) was added to each bottle. Prior to incubation at mesophilic temperatures (35 ± 2°C), the serum bottles were flushed with helium gas to provide anaerobic conditions. The BMP test was continued until the cumulative biomethane production reached a plateau (after ~20–25 days of incubation) and the daily biogas production was measured by the volumetric liquid displacement method using sodium hydroxide (120 g NaOH/L) to capture carbon dioxide.

### Design and Operation of the Upflow Side-Stream AnMBR

**Figure 1:**
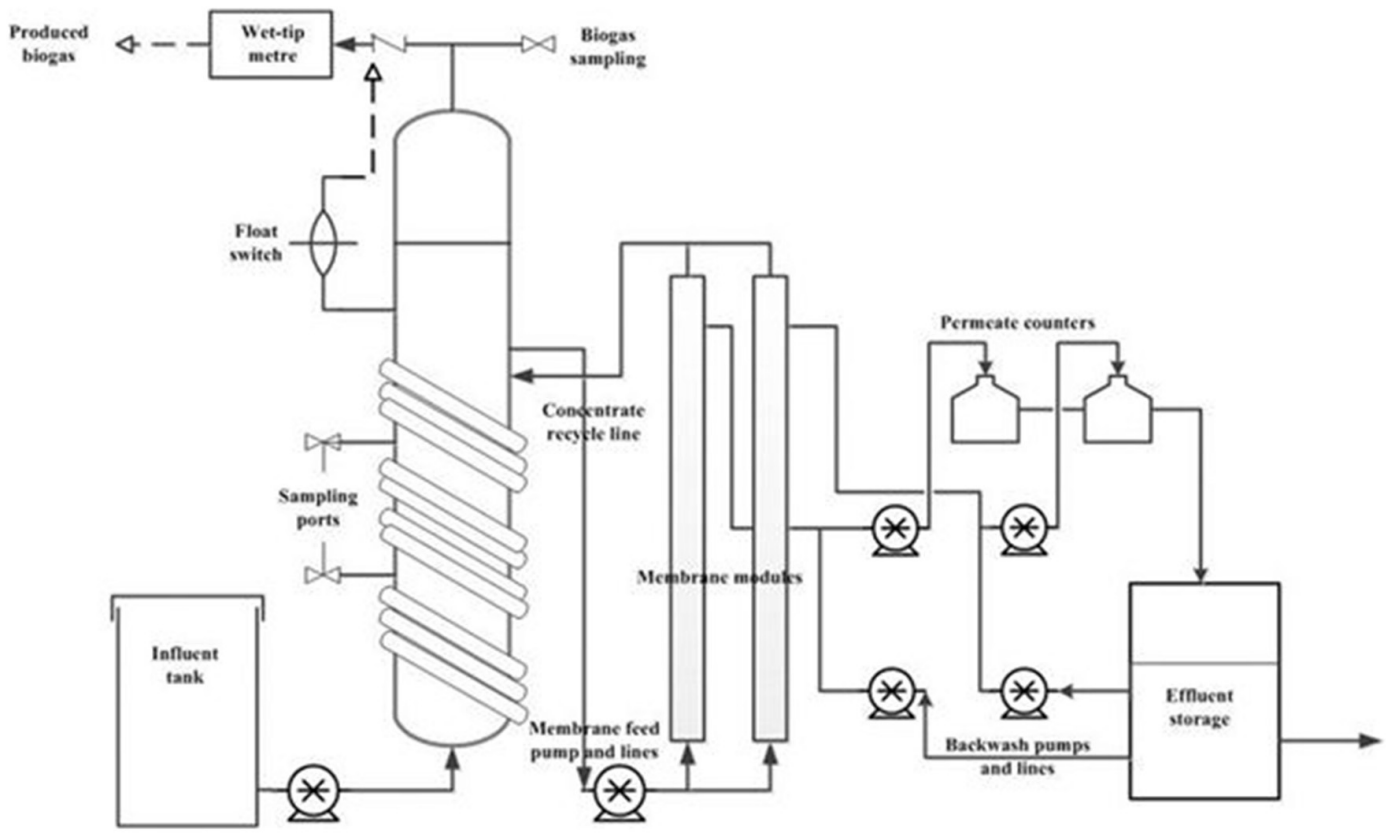
Schematic diagram of the side-stream AnMBR experimental set-up used in this study for the anaerobic treatment of diluted FW.

A laboratory scale AnMBR consisting of an upflow anaerobic bioreactor coupled to two side-stream ultrafiltration membrane modules connected in parallel was used for this study (Figure 1). The total working volume of the bioreactor was 10 L with a 3 L headspace. The bioreactor temperature was kept at mesophilic temperatures (35 ± 2°C) by recirculating warm water through a coil wrapped around the column. Each of the membrane modules contained one 0.88 m × 8 mm ID polyvinylidene fluoride (PVDF) ultrafiltration (UF) tubular membrane (Norit X-Flow, F5385) with a mean pore size of 0.03 μm and filtration area of 0.066 m^2^. Synthetic FW was fed to the bioreactor using a peristaltic pump using different flow rates based on the required HRT. The spent digestate from the bioreactor was delivered to the membrane modules by a peristaltic pump with a cross flow velocity (CFV) of 0.1 m/s. The concentrate (rich in biomass) from the membrane modules was recycled back to the bioreactor. Membrane performance was monitored following Prieto et al. (2013) using Cole Parmer pressure transducers (Cole-Parmer Instrument Company, IL, United States) in the feed, concentrate and permeate lines, and an ONSET Weather Station data logger (ONSET Computer Corporation, MA, United States) (Prieto et al., 2013). Transmembrane pressure (TMP) was calculated following the EPA manual for membrane treatment (Pirnie and Allgeier, 2005). The membrane permeate flux was measured by an Arduino board connected to the data logger.

The AnMBR system was started with an HRT of 20 d (OLR of 0.3 gVS/L.d). When stable operation was achieved (as indicated by a constant volatile organic acid (VOA)/ partial alkalinity (PA) ratio, high COD removal efficiency (98%), and stable methane production), the HRT was reduced. After each step decrease in HRT, the bioreactor was allowed to reach stable operation before further lowering the HRT. HRTs of 10, 7, 5, 3, and 1 d corresponded to OLR values of 0.6, 0.86, 1.2, 2, and 6 gVS/L.d, respectively. The HRT and OLR were calculated based on the produced permeate volume from both membrane modules together.

Membrane flux was monitored throughout the experiment. The HRT was varied by increasing the duration of the membrane filtration cycles to increase the system throughput at higher HRTs. The filtration time required for a specific HRT was divided into 4 cycles throughout the day, with relaxation periods occurring between active filtration cycles. Four cycles were applied during all HRTs, with an exception for an HRT of 1 d where the filtration cycle was increased to 8 per day. The filtration cycle intervals were controlled by a timer connected to the permeate pump. Starting from an HRT of 5 d, backwashing was performed after each filtration cycle to reduce the membrane fouling. The backwash flux was 10 times higher than the filtration flux and the backwash cycle was controlled by a timer.

### Analytical Methods

Liquid samples (50–60 mL) from the bioreactor were collected approximately once in 2–3 days to prevent washout of bacterial biomass. Tedlar gas bags (1L) were used for sampling the biogas. Total solids (TS), total suspended solids (TSS), and volatile solids (VS) were determined following the standard methods (American Public Health Association, 2005). Total and soluble chemical oxygen demand (COD_tot_, COD_sol_), total nitrogen (TN), total phosphorus (TP), and total ammoniacal nitrogen (TAN) were analyzed with HACH test kits following the manufacturer's instructions (HACH, Loveland, CO, United States). Total alkalinity (TA) and partial alkalinity (PA) were calculated based on the volume of the consumed hydrochloric acid (0.1N) by titrating with it until pH 5.75 and 4.3, respectively (Ariunbaatar et al., 2015). Based on the TA and PA values, the volatile organic acid (VOA) alkalinity and VOA/PA ratio were calculated. Continuous biogas production from the AnMBR was measured by a wet-tip meter. Methane content was analyzed with a gas chromatograph (GC) unit by Agilent Technologies (Agilent 7820A) equipped with a thermal conductivity detector (TCD) and a 30-m J&W 113-3133 GS-CarbonPLOT, 0.32 mm diameter column (Agilent Technologies, Lexington, MA). All analyses were performed in duplicates.

## Results

### Seed Sludge and FW Characteristics

The pH, TS, VS, total and partial alkalinity, total ammoniacal nitrogen (TAN), total nitrogen (TN), and total phosphorus (TP) of the seed sludge were 7.7 (± 0.1), 18.7 (± 2.4) mg/L, 13.1 (± 0.1) mg/L, 4,389.7 (± 10.7) mg/L, 3,886.0 (± 11.6) mg/L, 396.7 (± 2.4) mg/L, 405.0 (± 18.7) mg/L, and 103.8 (± 7.0) mg/L, respectively. The TS of the synthetic FW was always in the range of 238.6–266.6 mg/kg, and >95% were volatile solids.

**Figure 2:**
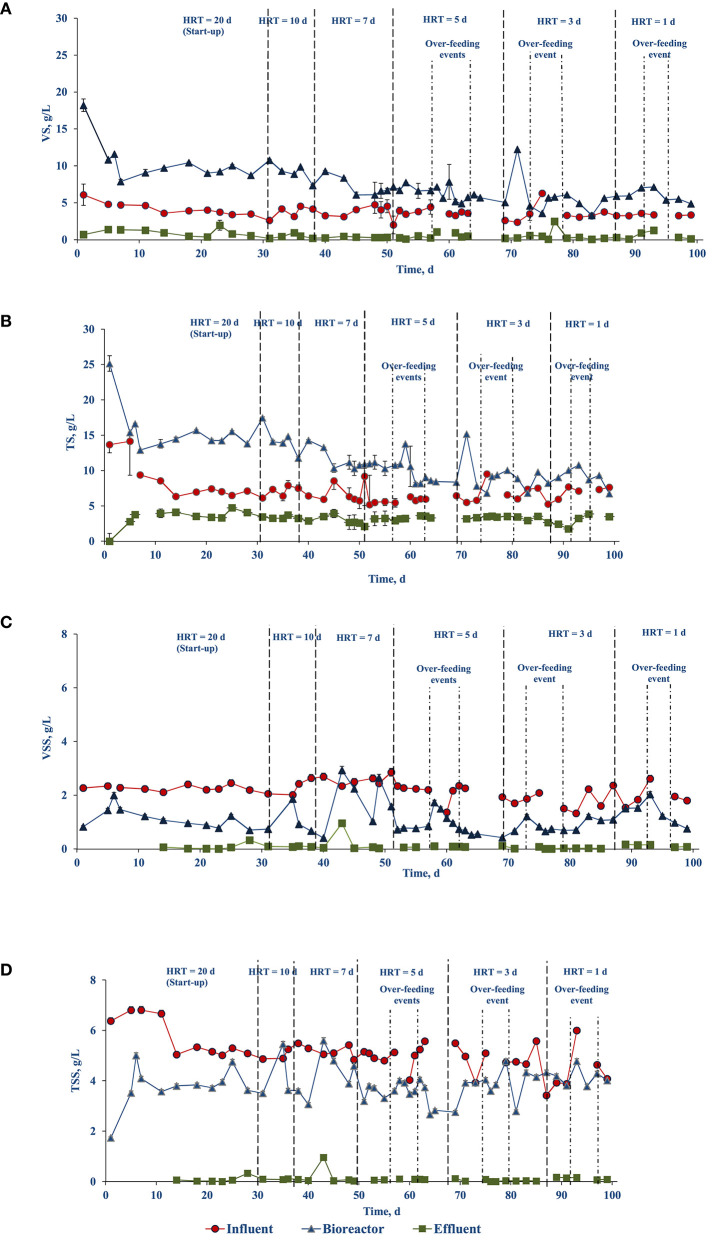
Evolution of the solids profile of the food waste treating side-stream AnMBR over time: **(A)** VS, **(B)** TS, **(C)** VSS, and **(D)** TSS.

Figure 2A shows the VS profile in the bioreactor and influent over time. At the start, the VS in the bioreactor and influent (feed) were 14.53 (± 0.43) and 6.09 (± 1.44) gVS/L, respectively. However, after 7 days of operation, the reactor VS was reduced to 9.08 (± 0.43) gVS/L. To keep the food to inoculum ratio 0.5 gVS/gVS, the influent VS concentration was reduced accordingly. After day 7 the VS in the influent was kept in the range of 3–4.7 mg/L, resulting in a reduction of the OLR, and thus a slight deviation from the designed OLR (0.6 gVS/L.d) described in section Design and operation of the upflow side-stream AnMBR. The influent TS, VS, TSS, and VSS were 6.68 (± 0.28), 3.66 (± 0.29), 5.09 (± 0.72), and 2.05 (± 0.36) g/L, respectively (Figure 2).

### Performance of the Upflow Side-Stream AnMBR

#### pH and Alkalinity

**Figure 3:**
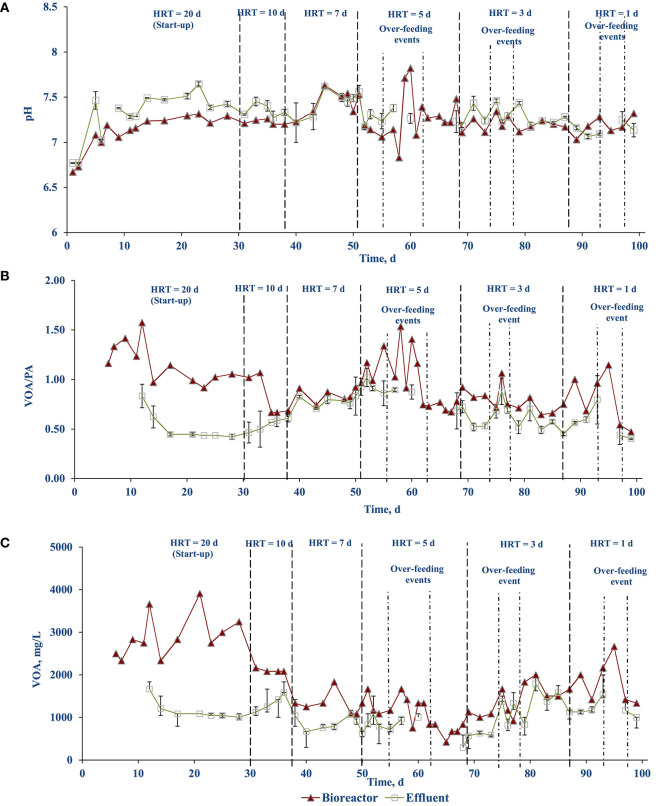
Stability profile of FW treating side-stream AnMBR: **(A)** pH, **(B)** VOA/PA ratio, and **(C)** VOA.

Figure 3A shows the pH of the AnMBR mixed liquor (bioreactor) and the effluent from the membrane modules during the operational periods. The pH inside the bioreactor was slightly lower than the effluent pH. As the solids content in the bioreactor sample could be interfering with the pH measurement, the measurement was taken on the soluble (centrifuged) fraction of the bioreactor content. When this test was preformed, there was no difference in the pH values between centrifuged and non-centrifuged samples. A possible explanation for the difference in bioreactor and effluent pH values could be the difference of carbon dioxide (CO_2_) partial pressure in the headspace, with the effluent having a lower CO_2_ concentration, yielding a higher pH value. This explanation is supported by the lower effluent VOA concentrations (Figure 3C).

The VOA/PA ratio of the bioreactor as well as the effluent was stabilized after 14–17 days at values of 0.86 and 0.49, respectively. Each time when the OLR is increased (including the unintentional OLR shock due to over-feeding events on days 58, 60, 76, and 95), the ratio increased immediately but stabilized after a while (Figure 3B). A similar trend can be observed with the VOA concentration (Figure 3C). This indicates the AnMBR system could handle the OLR shocks and recover quickly.

#### Nutrient (TN, TAN, and TP) Removal

**Figure 4:**
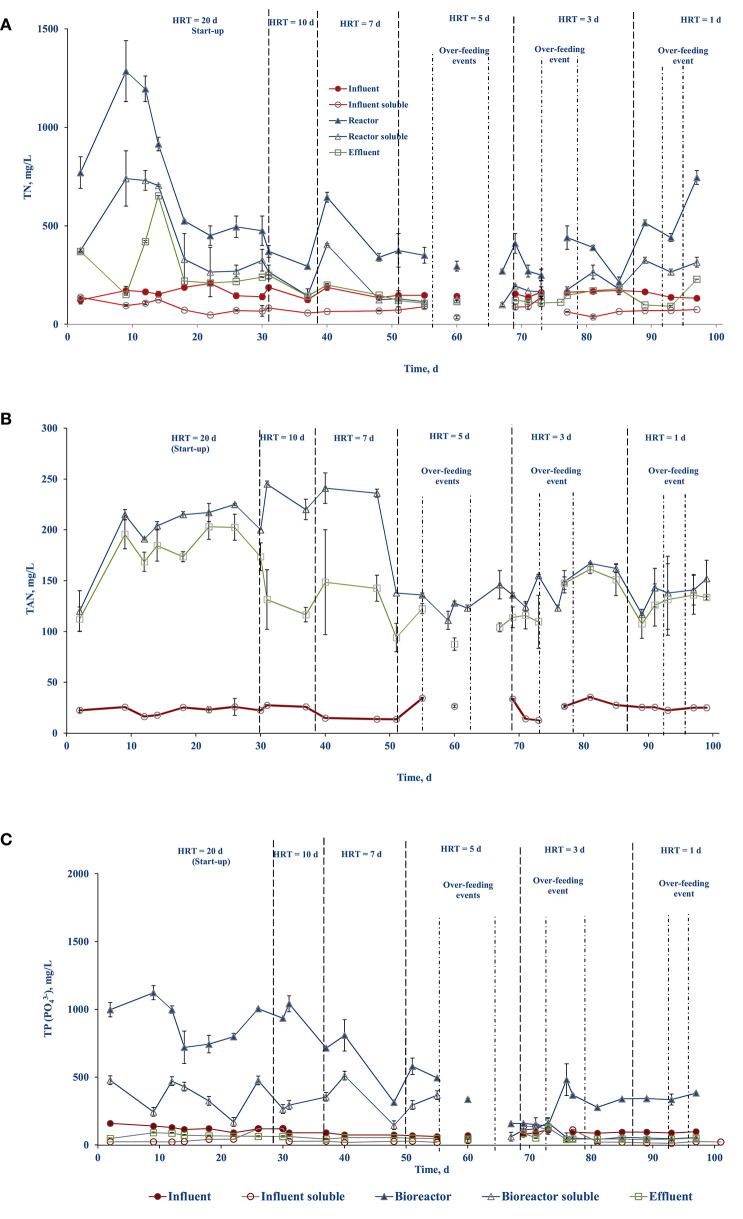
Nutrient profile in the different streams over the course of side-stream AnMBR operation: **(A)** TN, **(B)** TAN, and **(C)** TP.

The nutrient concentrations (TN, TAN, and TP) in the influent (feed), bioreactor, and effluent are presented in Figure 4. Figures 4A,B show the TN and TAN concentrations stabilized in the bioreactor and effluent after 15–20 d. The TN concentration of the influent averaged 157 (± 21) mg/L with an influent soluble concentration of 78.45 (± 26) mg/L. The TN concentration in the bioreactor and effluent during days 1–50 averaged 647 (± 316) and 269 (±141) mg/L, respectively, and between days 51 until the end averaged 370 (± 135) and 131 (± 37) mg/L, respectively (Figure 4A).

The TAN concentration in the bioreactor and the effluent were 210.75 (± 31.4) and 162.63 (± 30.7) mg/L, respectively, during days 1–50. Starting on day 51 until the end of the experiment, they lowered to 138.28 (± 15) and 122.80 (± 20.4) mg/L, respectively (Figure 4B). The higher TAN level explains the slightly higher pH during the day 1–50 period (Figure 4B).

The TP concentration in the bioreactor showed fluctuations with its concentration decreasing in the bioreactor over the course of operation (Figure 4C). The TP concentration averaged 850 (± 209) and 319 (± 131) mg/L during the start till day 50 and from day 51 until the end, respectively. The influent TP averaged 100 (± 25) mg/L. The high variability in TP in the bioreactor during the initial half of the study was due to its accumulation, where some fraction of the TP was bound in the microbial consortia and/or in a particulate fraction that was retained by the membrane. However, the TP concentration in the effluent was stable throughout the experiment and averaged to 59.61 (± 20.03) mg/L (Figure 4C). In general, the concentration of the nutrients in the bioreactor was higher than in the influent, indicating nutrient accumulation in the bioreactor mixed liquor.

#### Solids and COD Removal

Figure 2 shows the profile of the solid's concentration in the influent, bioreactor, and effluent. Throughout the experiment, the solids concentrations in the influent and effluent were relatively constant. The effluent TS, VS, and TSS concentrations were 3.31 (± 0.61), 0.55 (± 0.53), 0.08 (± 0.01) g/L, respectively, whereas VSS concentrations were below the detection limit. However, on day 43 the total suspended solids content in the effluent increased by 3 folds. Concomitantly, TS and VS concentrations in the bioreactor were, respectively, 14.29 (± 0.01) and 9.20 (± 0.03) g/L during the operation days 7–43, and were reduced to, respectively, 11.01 (± 0.27) and 6.75 (± 0.18) g/L during days 45–60 (Figures 2A,B). The TSS and VSS content in the bioreactor were reasonably constant at values of, respectively, 3.86 (± 0.62) and 1.08 (± 0.55) g/L. Moreover, the decrease of the TSS and VSS concentration in the reactor on days 64 and 81 was due to no feeding following over-feeding events.

**Figure 5:**
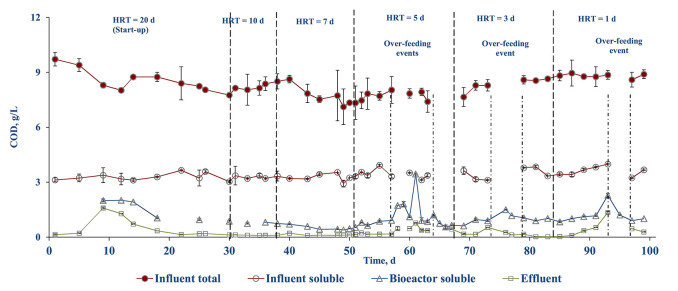
COD profile in the different streams during AnMBR operation.

The influent COD_tot_ and COD_sol_ concentrations were 8.24 (± 0.12) and 3.31 (± 0.05) g/L, while the COD concentration in the bioreactor varied depending on the OLR (Figure 5). When the OLR was increased (excluding the over-feeding events) during the stepwise HRT reduction, the COD_sol_ in the bioreactor increased yielding a higher COD concentration in the effluent (0.35–0.77 g COD/L) (Figure 5). However, the bioreactor COD_sol_ was reduced immediately after 2 days resulting in an effluent COD concentration of 0.03–0.15 g/L. The highest COD_sol_ concentration of 3.45 (± 0.13) g COD/L in the AnMBR mixed liquor was observed on day 61 after the loss of biomass (~3 L) and an over-feeding event. Nevertheless, the ultrafiltration membranes of the AnMBR were able to retain the remaining biomass in the system and recover its performance within a week. The first two over-feeding events resulted in an OLR shock, from which the AnMBR recovered quickly. Even during the unintentional over-feeding events on days 76 and 93, the AnMBR was able to recover in <2 days.

**Figure 6:**
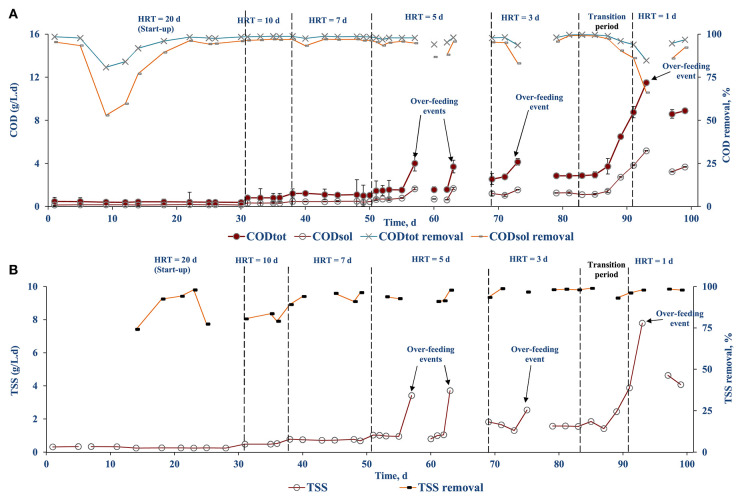
COD loading and removal efficiency of diluted FW fed AnMBR: **(A)** CODtot and CODsol and **(B)** TSS.

The COD removal efficiency was calculated based on the influent and effluent COD concentrations (Figure 6A). The COD removal combines both the biological process and membrane rejection of the AnMBR system. The overall COD_tot_ and COD_sol_ removal efficiencies were >97 and >95%, respectively. During the stepwise HRT decrement, the COD removal efficiency dropped by 3–5% and recovered after 2–3 days. The COD removal efficiency was not calculated during over-feeding events, i.e., operational days 57–60, 63–69, 76–79. During stable operation, >98% TSS removal efficiency was achieved. A similar decreased TSS removal efficiency was observed each time when the HRT was reduced (Figure 6B).

#### Biogas Production, Methane Yield, and Energy Generation

The BMP test of FW lasted for 25 days and reached a maximum of 472.15 (± 1.75) mL CH_4_/gVS_added_ (or 418.50 ± 1.55 mL CH_4_/g VS_added_ at STP). Due to several over-flowing and headspace leaking events the data from the wet tip meter was unreliable and the produced biogas volume could not be quantified during the initial operational days (HRT 20, 10, and 7 days). Therefore, the value obtained from the BMP test was used to calculate the biogas production from the AnMBR. Table 1 shows the average biogas production calculated based on the VS loading. During subsequent operation, the methane content in the biogas increased with increasing OLR and decreasing HRT (Table 1). The maximum methane content detected was 70.5 (± 3.5)% for the HRT of 1 d. The highest methane yield, 0.139 L CH_4_ / g COD (35.2% of the theoretical maximum yield at 35°C), was achieved at an HRT of 3 days, although methane yields at 5 d and 1 d HRTs were very close to this value as well, measured as 0.124 and 0.125 L CH_4_/g COD, respectively (Table 2).

**Table 1:** Biogas production and composition of the side-stream AnMBR at different HRT.

**HRT**	**VS_**added**_ (g/d)**	**Biogas (L/d)**	**% Methane in biogas**
20	1.97 ± 0.02	0.93	–
10	3.63 ± 0.05	1.71	–
7	5.72 ± 0.25	2.70	–
5	11.25 ± 0.69	5.31	49.6
3	13.07 ± 0.31	6.17	67.6
1	30.02 ± 0.05	14.17	70.5

**Table 2:** Methane yield, energy production, and estimated electrical energy revenue for the present study.

**HRT (d)**	**Biogas production (L/d)**	**Methane content (%)**	**Methane production (L/d)**	**COD consumed (g COD/d)**	**Methane conversion (mL/g COD consumed)**	Q (L/d)^*^	Energy content (kJ/d)^**^	**Energy (MJ/m^**3**^)**	**Electricity (kWh/m^**3**^)**	**Heat, (Btu/m^**3**^) treated**	**Possible electrical energy revenue (EUR/m^**3**^)**
20	0.9	–	–	4.3	–	0.5	–	–	–	–	–
10	1.7	–	–	8.2	–	1.2	–	–	–	–	–
7	2.7	–	–	11.2	–	1.7	–	–	–	–	–
5	5.3	49.6	2.6	21.2	124	1.9	94.3	48.9	5.7	20,096.3	0.67
3	6.2	67.6	4.2	30.0	139	3.5	149.3	42.9	5.0	17,650.2	0.59
1	14.2	70.5	10	80.0	125	11.4	357.6	31.4	3.7	12,904.8	0.43

* real flow rates based on actual HRT

** based on 35.8 kJ/L CH_4_ (lower heating value).

Calculated based on Combined Heat and Power Efficiency Values: CHP Electrical Output- 42.2%, CHP Thermal Output- 43.4% and CHP loss- 14.4% and Electricity cost (Non-Household): EU avg. (EUR/kWh)-0.1173.

The highest energy content of the collected biogas per m^3^ of food waste treated was achieved at 5 d HRT at 48.9 MJ/m^3^ treated, whereas the energy content of biogas at 1 d and 3 d HRTs were 31.4 and 42.9 MJ/m^3^ treated, respectively (Table 2). These findings suggest that while it is possible to bring down the HRT of the system to 1 d with no significant adverse effects on the treatment performance, operation at 3 d HRT ensures maximum methane yield, while operation at 5 d HRT ensures highest energy output per m^3^ treated. At 3 d HRT, the energy content of the biogas amounted to 5.0 kWh/m^3^ as electricity output, and 17,650 Btu/m^3^ as heat output if the biogas is processed via a combined heat and power system with electrical and thermal efficiencies of 42.2 and 43.4%, respectively. For a 5 d HRT system, the energy output would be 5.7 kWh/m^3^ as electricity and 20,096 Btu/m^3^ as thermal output.

#### COD Balance

**Figure 7:**
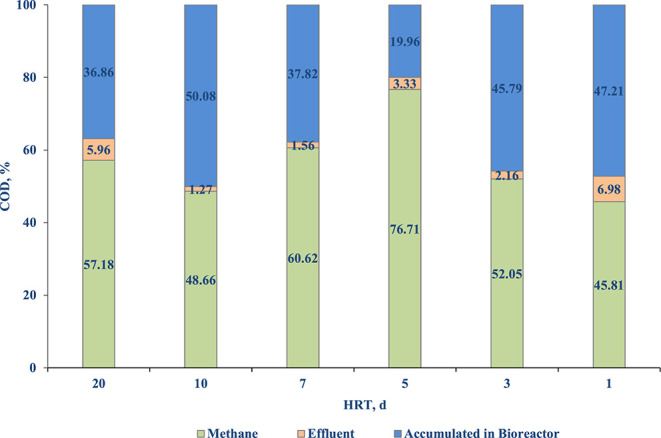
COD balance of the side-stream AnMBR treating diluted FW.

The COD balance was calculated based on the influent COD, biomethane production and effluent COD. The biomethane production was converted to COD using the theoretical conversion of 0.395 L/g COD (0.35 L/g COD at STP). The COD balance for different HRTs is shown in Figure 7. The COD to methane conversion increased with decrease in HRT, reaching a highest conversion of 76.71% at an OLR of 1.84 g COD/L.d and HRT of 5 d. During this period, a minimum COD accumulation of 19.96% was observed. After this period the methane conversion ratio reduced to 52.05 and 45.81% at an OLR of 3 and 8.65 g COD/L.d, and HRT of 3 and 1, respectively.

#### Membrane Performance

**Figure 8:**
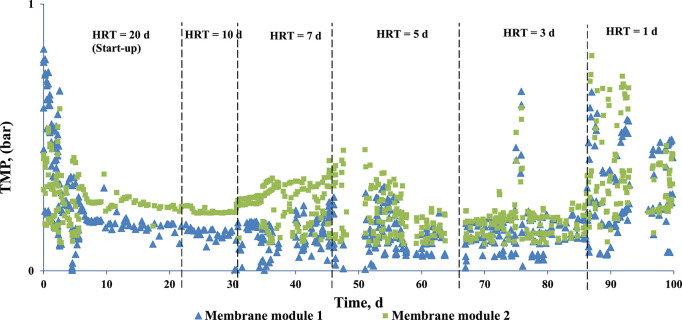
TMP profiles of the side-stream membrane modules of the AnMBR.

Table 3 gives the estimates of the membrane performance parameters permeate flow rate and flux at the different HRT (proposed and actual) investigated. In the present study, the membranes did not show any failure throughout the course of the 100 days study period of 100 days and have not been changed. During the start-up period, the average flux and TMP of the two membranes were 7.18 LMH and 0.28 bar, respectively, with a calculated HRT of 18.5 d (Table 3, Figure 8). The TMP of 0.28 bar is a result of the initial high TMP that was observed during the start of the membrane process. When the HRT was reduced from 20 to 10 d, the TMP decreased to 0.20 bar (Figure 8). The operational process, i.e., membrane extraction conducted at an operational interval of four per day, did not show impact on the TMP or the flux during the HRT decrement from 20 to 10 d (Table 3, Figure 8). However, the HRT reduction from 10 to 7 d resulted in an increase in TMP and reduction in flux, suggesting membrane fouling due to extended operation of the membrane (Table 3, Figure 8). To alleviate the effect of fouling, the HRT decrease from 7 to 5 d was coupled with a backwash cycle. TMP reduced significantly (from 0.24 to 0.18 bar) and the flux was stabilized at 12.26 LMH (Table 3, Figure 8). Both the flux (13.66 LMH) and TMP (0.20 bar) were relatively constant during the HRT decrease from 5 to 3 d as well. When the HRT was decreased from 3 to 1 d, the planned flux and TMP were not achieved as the membrane performance was not stable. This resulted in a transition phase where the average flux and TMP during this period was 9.53 LMH and 0.32 bar, resulting in an actual HRT of 2.41 d instead of the planned 1 d (Table 3, Figure 8). This unplanned transition period lasted for 8 days and was probably caused by the intensive increase in OLR, creating an accumulation of solids which further fouled the membrane, thus increasing the TMP and decreasing the flux. Nevertheless, after 8 days, the system was able to overcome this issue and a stable operation with an actual HRT of 0.88 d and a flux of 11.4 LMH was obtained.

**Table 3:** Membrane performance characteristics (Flux and TMP) of the side-stream AnMBR treating diluted FW at different HRT.

**HRT (d)**	**Q (L/d)**	**Flux (LMH)**	**TMP (bar)**	Q* (L/d)	HRT* (d)
20	0.50	7.18	0.28	0.54	18.50
10	1.00	8.26	0.20	1.21	8.29
7	1.43	7.71	0.24	1.66	6.04
5	2.00	12.26	0.18	1.93	5.18
3	3.33	13.66	0.20	3.48	2.88
Transition	10.00	9.53	0.32	7.27	2.41
1	10.00	14.21	0.28	11.40	0.88

HRT, Theoretical hydraulic residence time; HRT*, Actual hydraulic residence time; Q, Theoretical flow rate; Q*, Actual flow rate; TMP, Transmembrane pressure.

## Discussion

### AD of Diluted FW in AnMBR

This study showed that the AnMBR could treat diluted FW (7% solids content) and produce biogas with a high methane content at an HRT of 1 d. The OLR is a key parameter for biogas production through AD as process failures can occur at high organic loading rates (OLRs) (Kleyböcker et al., 2012). HRT has a significant effect on biogas production: a longer HRT favors higher biogas production along with higher methane content (Shi et al., 2017). The study by Cheng et al. (2018) reported that for an OLR of 9.72 g COD/L d, the biogas production rate was maximum but decreased when the OLR was further increased. However, in the present study, the biogas production increased with OLR loading as the maximum loading rate applied was 8.85 (± 1.6) g COD/L d (Figure 6A).

The biogas production increased during the HRT decrements due to the addition of VS_added_ (Table 1). The maximum methane percentage in biogas was calculated to be 70.1% which was very well in the range reported in the literature (73%) for FW degradation through AD (Zhang et al., 2007). The lowest accumulation of COD within the reactor was obtained at 5 d HRT, which is also when the highest COD conversion to methane was observed (Figure 7). The maximum biogas production achieved in the present study was 14.17 L/d for an OLR of 8.85 (± 1.6) g COD/L d (Table 1). Wijekoon et al. (2011) reported that the biogas generation increased from 15, 20 and 35 L/d when the OLR was increased from 1 (± 0.1), 8.1 (± 0.3) and 12.0 (± 0.2) kg COD/m^3^d. The low biogas production that was observed was due to the diluted influent feed that was used in this study. It was proposed that the biogas production can be improved by further utilizing the COD trapped inside the sludge using a second digester (Mota et al., 2013). The proposed process flow design (anaerobic–partial nitrification–annamox) by Wan et al. (2016) for COD capture and biogas production can even further maximize the treatment and utilization of the substrate for energy generation.

The AnMBR system showed high COD_tot_ and COD_sol_ removal efficiencies, i.e., >97 and >95%, respectively (Figure 6A). The lower biological COD removal efficiency in the start-up period that was observed can be explained by the loss of biomass (~2–3 L) due to malfunctioning of the pumps (Figure 6A). This, however, recovered and stabilized after 10 days. The results are very well in agreement with the literature on COD removal by AnMBRs with a minimum of 67% been reported for wastewaters from the food industry (Dvorák et al., 2016). The COD removal efficiency typically depends on the OLR of the reactor (Balcioǧlu et al., 2021). From the literature it can be gathered that AnMBRs can operate at varied OLRs, with low OLR providing better pollutant removal at higher biomass concentration in the reactor and high OLRs favoring stabilized treatment efficiency with active biomass retained by the membranes (Balcioǧlu et al., 2021). Wijekoon et al. (2011) reported that the COD removal efficiency increased with OLR reaching a maximum removal efficiency of 80% at an OLR of 8.0 (± 0.3) g COD/L d, but decreased to almost 60% at an OLR of 12.0 (± 0.2) g COD/L d when treating a synthetic wastewater using a thermophilic AnMBR. The observed decrement was later attributed to the lack of microbial concentration in the reactor. Nagao et al. (2012) studied the effect of OLR with active biomass where the active biomass increased to 8.5 × 10^10^ cells/g TSS at an OLR up to 7.4 gVS/L d, but a further OLR increase resulted in a reduction of cell density to 5.0 × 10^10^ cells/g TSS, and hence an overall reduced biological activity. In the literature there is the sustainable OLR concept where the AnMBR performance may decrease if the sustainable OLR value is exceeded (Wei et al., 2014). In the present study, a maximum OLR of 6 gVS/L d was used, which is well within the sustainable OLR value as the COD removal efficiency and performance were not affected up to that OLR (Figure 6A).

The stability of the anaerobic digestion process can be monitored through operational parameters such as the pH and VOA/PA ratio (Ariunbaatar et al., 2015). Throughout the course of AnMBR operation an average pH of 7.2 (± 0.2) was maintained (Figure 3A). This was well within the optimum pH range 6.8–7.2 where the process can tolerate up to pH 8.0 (Cioabla et al., 2012). The VOA/PA ratio of the reactor averaged at 0.86. During stepwise OLR increase, the VOA/PA ratio increased, indicating indigestion (Figure 3B). The reactor, however, recovered after a few days and operational stability was achieved. It is reported in the literature that the AD process is regarded as stable when the volatile fatty acids (VFA)/total alkalinity (TA) ratio ranges between 0.23 and 0.3 (Chen et al., 2020).

To further illustrate the significance of the study, Table 4 compares similar research and illustrates the advantage of the side-stream AnMBR system used in this study. For instance, this study maintained a stable operation at an OLR of 8.85 (± 1.6) g COD/L.d with more than 97% COD removal, while Amha et al. (2019) reported a stable operation at OLR of 10 g COD/L.d with 99% COD removal rate for the treatment of FW. However, Amha et al. (2019) used a two-stage AnMBR system with separate acidification phase and HRT of 3 days, which is three-fold higher HRT of this study. Moreover, the AnMBR system treating FW slurry or meat-processing wastewater were able to achieve a HRT of 1 day, but the OLR was also lower at 1.014 (± 0.066) g COD/L.d (Moñino et al., 2017) or 3.2 g COD/L.d (Galib et al., 2016), respectively. Similar methane yields and COD removal efficiencies at high OLR have thus far only achieved at relatively higher HRT of 5–15 days with the most used one-stage or two-stage CSTR systems (Paudel et al., 2017; Bi et al., 2020).

**Table 4:** Comparison of other reported studies with low HRT treating different FW.

**Feed (type of FW)**	**Feed content (TS, VSS, COD)**	**Reactor type**	**OLR**	**Lowest HRT**	**Temperature**	**Methane production (yield/percentage)**	**Effluent content (TS, VSS, COD)**	**References**
Lab made FW	3.78 (± 0.14) g VS/L 8.24 (± 0.12) g COD/L	AnMBR	8.85 (± 1.6) g COD/L d 6 g VS/L.d	1 day	Mesophilic (32–39°C)	0.125 L.CH_4_/g COD_removed_ 70% methane	> 97% COD removed 0.28 g COD/L 0.16 (± 0.01) TSS	Present study
Lab made FW	22.06 (± 0.16)%VS	CSTR	0.9 g VS/L.d	20 days	Mesophilic (32–37°C)	0.3379 L.CH_4_/g VS_added_ 40–50% methane	N/A	Ariunbaatar et al., 2015
		2-stage CSTR				0.3821 L.CH_4_/g VS_added_ 50–60% methane	N/A	
Lab made FW with trace elements (Fe, CO, Ni) addition	38.9 (± 4.3) g VS/L	AnMBR	4.37 (± 0.65) g COD/L/d	15 days	Mesophilic	0.42 L.CH_4_/g TS_fed_ 59.3 (±1.2)% methane	0.37 (± 0.14) g COD/L	Cheng et al., 2020b
Lab made FW with trace elements (Fe, CO, Ni) addition	43.03 (± 0.83) g VS/L. 73.67 (± 3.1) g COD/L	AnMBR	9.72 g COD/L/d	7.5 days (at HRT 5 the operation became unstable)	Mesophilic	280 (± 0.02) L.CH_4_/kg COD_removed_ 58.9 (± 0.3)% methane	80% COD removed 0.25–0.51 g/L	Cheng et al., 2018
		CSTR	6.59 g COD/L.d	N/A		N/A	75–78% COD removed 14–20 g/L	
Synthetic FW	72.9 (± 3.8) g COD/L	AnMBR	6.00 (± 0.59) g VS/L/d (at OLR of 9.0 g VS/L.d acidification occured)	7.5 days (at HRT 5 the operation became unstable)	Mesophilic	0.48 (± 0.04) L.CH_4_/g VS_fed_ 58.9 (± 0.3)% methane	N/A	Cheng et al., 2020a
FW collected from canteen	6 (± 0.3)% VS 74.6 (± 3.5) g COD/L	CSTR	15 g VS/L.d	5 days (at HRT 4 reactor became unstable)	Mesophilic (37°C)	0.126 (± 0.22) L.CH_4_/g VS_fed_ 56 (±1)% methane	N/A	Bi et al., 2020
Processed FW mixed with creamery WW	122 (± 7) g COD/L	Single stage-AnMBR	2.5–15 g COD/L.d (2-stage system inhibited at OLR of 15 g COD/L.d)	3 days	Mesophilic (37°C)	0.32 (± 0.06) L.CH_4_/g COD fed obtained at OLR of 2.5 g COD/L.d	99% COD removed	Amha et al. (2019)
		Two-stage AnMBR				0.33 (± 0.02) L.CH_4_/g COD_fed_ obtained at obtained at OLR of 3.5 g COD/L.d		
Pretreated household FW slurry	0.444 (± 0.057) g COD/L	AnMBR	1.014 (± 0.066) g COD/L.d	1 (± 0.2) day	Mesophilic (25–28°C)	239.6 L.CH_4_/d 74.7% methane	0.0519 g COD/L 94% COD removed	Moñino et al., 2017
			0.717 (± 0.078) g COD/L.d	1 (± 0.1) day		124.2 L.CH_4_/d 62% methane	0.0257 g COD/L 97% COD removed	
Meat-processing wastewater	4.398 (± 0.305) g CODt/L	AnMBR	3.2 g COD/Ld	1 day	Ambient temperature (24 ± 2^O^C)	0.18 (± 0.08) L.CH_4_/g COD_removed_	88–95% COD removed	Galib et al., 2016
Household FW	4.78 (± 041)% VS 127 (± 5.17) g COD/L	2 stage CSTR	106 g VS/L.d	0.33 day (acidogenic reactor)	Mesophilic (35°C)	1.35 (± 0.08) L/d	18.66 (± 1.63)% COD removed	Paudel et al., 2017
			1.76 g VS/L.d	15 days (methanogenic reactor)		23.2 (± 2.26) L/d 0.188 L.CH_4_/g COD 60 (± 2.75)% methane	50.4 (± 1.70)% COD removed	

N/A, Not Applicable.

The energy generated (electricity) in a combined heat and power system can be sold to the grid for revenue, to offset the capital and operating costs of the system. In the European Union, the average cost of non-household electrical energy for EU-27 countries is 0.1173 EUR (Eurostat, 2020). This corresponds to possible revenues of 0.67 and 0.59 EUR/m^3^ for 5 and 3 d HRT operation, respectively, if all of the electrical energy can be supplied to the grid.

### Membrane Separation

The main goal of the membrane system is to retain biomass and suspended solids in the bioreactor and establish stable operating conditions for anaerobic digestion. In the present study, the PVDF tubular membrane was successful in establishing the process and assisted in removal of VS and TS (Figure 2). VS was almost completely (93%) removed [reduced to 0.53 (± 0.03) g/L] whereas total solids had a removal efficiency of 71.5% [reduced to 3.26 (± 0.07) g/L] (Figures 2A,B). In treatment of sugar vinasse using a two stage AnMBR, volatile total solids were completely removed [removal efficiency of 93 (± 2.0)%], while total fixed solids remained unchanged [removal efficiency of 5.1 (± 26.5)%] after membrane filtration (Mota et al., 2013). The effluent COD in the present study was lower than the COD in the reactor, indicating that the membrane was able to separate part of the soluble organics (7%) by the cake layer formed on the membrane (Figure 5).

The high TMP of the membranes during the start of the experiment (Figure 8) can be attributed to the high initial flux which results in attachment of foulants on the membrane. This however reduces before reaching a steady flux and TMP. The TMP profile (Figure 8) shows that with every stepwise decrement in HRT, the TMP initially increased and stabilized, implying membrane fouling. The fouling in the present system could be a result of either organic or inorganic compounds present in the system. Organics compounds (such as proteins) and microorganisms attach onto the membrane surface, resulting in biofouling (Nguyen et al., 2012). Inorganic compounds such as struvite and dipotassium ammonium phosphate formed in the system can also foul the membrane (Ozgun et al., 2013). Membrane properties play a significant role in struvite precipitation (Ozgun et al., 2013). The effect of fouling more pronounced when the HRT was decreased from 10 to 7 d, which resulted in the requirement of backwash. An et al. (2009) reported that a decrease in HRT from 10 to 5.5 h resulted in a decrease in solids removal capacity by a UASB reactor, thus inducing fouling of the membrane. The choice of operational flux is important in fouling management. Operating the system below the critical flux is an effective approach to control fouling (Ozgun et al., 2013). Increasing the flux from 10 to 12 LMH in an anaerobic submerged membrane bioreactor treating municipal wastewater resulted in unstable operation due to high fouling rates (Martinez-Sosa et al., 2011).

### Nutrients Removal and Recovery

The nutrients (TN, TAN, and TP) concentrations in the bioreactor were higher than the influent throughout the course of operation (Figure 4). The TN removal in biological systems involves nitrification and denitrification, where ammonium is converted to nitrate (through oxidation) which is subsequently reduced to nitrogen gas (Tchobanoglous, 2014). Oxidation of ammonium requires aerobic conditions while the AnMBR system provides only anaerobic conditions. Therefore, it is expected that the AnMBR system will be less effective in removal of TN as the conditions favorable for ammonium oxidation are limited (Figure 4). This has been well-supported in the studies by Wijekoon et al. (2015) and Song et al. (2018). The TN removal efficiency was reported to be negligible or <20% in an AnMBR treatment process (Wijekoon et al., 2015). In an AnMBR coupled to a membrane distillation hybrid system, a 10–30% of TN removal efficiency was reported (Song et al., 2018). In the present study, during the first 50 days of operation, the TN removal was negligible, and, in some cases, the effluent had higher TN concentrations than the influent (Figure 4A). This can be attributed to the interstitial nitrogen release by the seed sludge and the FW in the bioreactor, which may have been discharged into the effluent. However, during the second half on the operation, the TN concentration in the effluent decreased and reached a maximum removal efficiency of 16.5 (± 3)% with respect to the influent (Figure 4A). The TN removal efficiency with respect to the bioreactor was estimated to be 61.5 (± 3)% (Figure 4A). This could be a result of the conversion of nitrogen to sludge as uptake by the MLSS and membrane separation of this colloidal nitrogen present in the reactor (Kong et al., 2020).

The TAN concentration plays a vital role in anaerobic digestion (Rajagopal et al., 2013). High ammoniacal-nitrogen concentrations result in process instability, leading to lower biomethane production, or even process failure (Rajagopal et al., 2013). The inhibition arises at varying concentrations based on the substrate and the operational conditions used. The IC_50_ of TAN is around 3,800 mg/L (Ariunbaatar et al., 2015). In the present study the average TAN value in the bioreactor was 175 mg/L (Figure 4B), which was much lower than that IC_50_ inhibition value. The TAN concentration is also critical in maintaining the buffering capacity of the bioreactor (Ariunbaatar et al., 2015). Once the buffering capacity is consumed, the bioreactor pH will start to decrease by the accumulating VFAs and can result in process failure due to acidification (Ariunbaatar et al., 2015). An overall TAN removal efficiency of 17.3 (± 5)% was observed throughout the experiment with respect to the bioreactor (Figure 4).

The TP concentration in the bioreactor was higher than in the influent (Figure 4) and almost 37.5 (± 3)% of TP was estimated to be present in the bioreactor in soluble form. The overall phosphorus removal efficiency of the side-stream AnMBR with respect to the influent was estimated to be 40.39 (± 5)%. The phosphorus removal in the present system is likely due to chemical precipitation. The presence of the TAN in the bioreactor supports the formation and precipitation of struvite (MgNH_4_PO_4_.6H_2_O) or dipotassium ammonium phosphate (K_2_NH_4_PO_4_), which contribute to the phosphorus removal (Ozgun et al., 2013).

## Conclusion

The present study demonstrates the applicability of a side-stream AnMBR for the treatment of diluted FW using wet anaerobic digestion (solids content 7%). A typical HRT of 20 d was successfully reduced to 1 d in 100 d. The biological part of the system was fully stabilized after more than 2 weeks, and it was able to convert 50–76% of the influent COD into biogas with up to 70% methane content. Additional COD removal was performed by the membrane filtration process, making the COD removal efficiency of the whole system > 97%. Moreover, > 8% of the influent total suspended solids (TSS) was removed. The TN and TP removal efficiencies were estimated to be around 16.5 (± 3) and 40.39 (± 5)%, respectively. Furthermore, the energy output through biogas production indicates the economic feasibility of the side-stream AnMBR concept in treatment of diluted FW at low HRTs.

## Data Availability Statement

The raw data supporting the conclusions of this article will be made available by the authors, without undue reservation.

## Author Contributions

JA, RB, and OO designed and conducted the experiments, analyzed the results, and drafted the manuscript. HR analyzed the results and drafted the manuscript. GE, PL, and DY supervised the work and gave inputs during the development of the manuscript. All authors contributed to the article and approved the submitted version.

## Conflict of Interest

The authors declare that the research was conducted in the absence of any commercial or financial relationships that could be construed as a potential conflict of interest.
